# Asepsis vs. Antisepsis in the Treatment of Accidental Injuries

**Published:** 1891-01

**Authors:** B. F. Church

**Affiliations:** Austin, Texas


For Daniel's Texas Medical Journal.

### ASEPSIS VS. ANTISEPSIS IN THE TREATMENT OF ACCIDENTAL INJURIES.

BY B. F. CHURCH, M. D., AUSTIN, TEXAS.

Read before the Austin District Medical Society, Dec. 18, 1890.

IT IS not the object of this paper to describe the various methods of aseptic or antiseptic surgery, but to call attention to a discipline of supreme importance in the management of accidental or gunshot wounds, viz: the *prevention of contamination*, and especially to *protest* against *probing*.

According to modern science the idea of spontaneous decomposition is as unnatural as spontaneous generation, and it is well known now that three factors are necessary for putrefactive decomposition of an albuminous or albuminoid substance. 1, *moisture*; 2, *warmth*, and 3, the presence of micro-organism or fungi, known as *bacteria* and *micrococci*. Absence of any one of these factors will prevent decomposition. This is practically demonstrated by the preservation of meats and fish by either drying, freezing, or hermetically sealing them from the organisms of the air. A bloody injury—if the wound is clean—furnish all the conditions necessary for suppuration, except the fungi. Oozing blood and lymph is a perfect pabulum and a most excellent medium for the growth and development of spores or seeds of fungi that float in the air of all habitable localities.

Bergman's sepsin, or ptomains of the French authors, are the products of a fermentation process set up by the fungi and is highly poisonous, causing local inflammation, septic fever, etc.

The dangers of a septic wound does not end here, for the *fungi* themselves may invade the living tissues, or be carried by the lymph channels and veins into the general circulation, causing secondary deposits and metastases.

These results are oftentimes learned after bitter experiences. Unfortunately many surgeons are not unlike some of the old accoucheurs,—who very thoroughly disseminated septicæmia amongst their lying-in patients,—do not stop to study the cause or to consider that loss of limb or life can absolutely be prevented in a majority of cases by thorough ascepticism—which is purely a preventive mode of treatment. An ounce of prevention is certainly worth several pounds of cure in these cases,—more properly speaking an attempt at cure,—for too often the injury is irreparable.

A large proportion of incised and lacerated wounds, compound fractures and all gunshot wounds—with rare exceptions, as pointed out by Esmarch—are aseptic and require no disinfection.

The first object of a surgeon, upon taking charge of a fresh case of a bloody injury, should be to strenuously guard against septic cantomination.

The wound, under no condition, should be handled unless there is unmistakable evidence that it contains filth. By avoiding suppuration, almost a complete restitution of the normal conditions will take place in a great majority of cases, while with suppuration, the gravity of a given injury is enormously increased. On this subject Geista says: "It may be safely assumed that an examination by probing or digital exploration performed on the filthy floor of a public place or on the street pavement, even by the most experienced surgeon, cannot be and *is not* cleanly aseptic. It is extremely dangerous, unnecessary, hence culpable." Bergman's experience during the Russo-Turkish war has showed that most gunshot wounds are aseptic, and that, with the exception of those cases where shreds of soiled clothing or gun-wads were carried by the projectile into the bottom of the wound, healing *without* suppuration can be confidently expected if the wound is not infected by meddlesome and uncleanly surgery.

In this connection the observations of Reyher should not be overlooked, as they show the vast gains modern surgery has made over the older plan of treatment of gunshot fractures.

Gunshot fracture of the knee joint was formerly considered an indication for immediate amputation. Rayher treated eighteen fresh cases aseptically, that is by simply cleansing and disinfecting the skin about the wound and occluding the same by an antiseptic dressing. When the wound was gaping or there was ground to suspect the entrance of dirt or shreds of clothing into bullet track, dilitation, irrigation, and extraction of the foreign body with subsequent drainage, before the wound was sealed up. Of these eighteen cases *fifteen* recovered, with movable joints, *83.3% of recoveries*.

Of nineteen that came under his care, several days after the reception of the injury, with well-established suppuration, *eighteen died*, and *one* recovered with a *stiff joint*; in spite of an energetic antiseptic treatment by incisions, drainings, and irrigation. There was a mortality of *85%*.

Generally speaking, a safe rule to follow is never to probe a gunshot wound, even if the ball be lodged just under the skin; it is far safer to refrain from removing it until the channel cut by it had healed. The bullet soon becomes encysted and will do no harm, unless, possibly, it presses upon some important nerve trunk. The idea entertained by a few, that there is danger of lead poisoning if the ball remains, is too absurd to admit of argument.

